# The Role of Neutrophil, Monocyte and Macrophage Calprotectin and S100A12 in the Fibrotic Process

**DOI:** 10.3390/biom16040553

**Published:** 2026-04-09

**Authors:** Nora Elemi Regino-Zamarripa, Ana Sofía Burciaga, Moisés Bocanegra-Mondragón, Alfredo Cruz-Lagunas, Ángel Camarena, Luis Jiménez-Alvarez, Remedios Ramírez, Gustavo Ramírez-Martínez, Joaquín Zúñiga

**Affiliations:** 1 Laboratorio de Inmunobiología y Genética, Instituto Nacional de Enfermedades Respiratorias Ismael Cosío Villegas, Mexico City 14080, Mexico; 2 Tecnológico de Monterrey, Escuela de Medicina y Ciencias de la Salud, Mexico City 14380, Mexico; 3 Tecnológico de Monterrey, Posgrado en Ciencias Biomédicas, Escuela de Medicina y Ciencias de la Salud, Mexico City 14380, Mexico; 4 Programa de Ciencias Químico-Biológicas, Instituto Politécnico Nacional, Escuela de Ciencias Biológicas, Mexico City 11350, Mexico; 5 Facultad de Ciencias, Universidad Nacional Autónoma de México, Mexico City 04510, Mexico

**Keywords:** S100 proteins, calgranulins, calprotectin (S100A8/A9), fibrosis, proinflammatory process, neutrophils, monocytes, macrophages

## Abstract

Fibrosis is a progressive and irreversible mechanism affecting any organ. During tissue injury, fibroblast activation is necessary for wound healing but the uncontrolled accumulation of fibrotic tissue leads to local organ damage. The fibrotic process involves the excessive accumulation of extracellular matrix components and inflammatory mediators. Since sustained inflammation precedes fibrosis, the involvement of immune cells, like neutrophils, monocytes and macrophages, is crucial to elucidate its pathogenesis. These immune cells release proinflammatory cytokines and chemokines, and also proteins that act as fibroblast proliferation mediators, such as the S100/calgranulins subgroup, comprising S100A8, S100A9, and S100A12 proteins. Moreover, a homodimer of S100A8 binds to a homodimer of S100A9 forming the heterodimer S100A8/A9, called calprotectin, which is abundant in the cytosol of neutrophils during immune activation. Although calprotectin (S100A8/A9) is the most predominant form, calgranulins S100A8 and S100A9 have independent functions of calprotectin (S100A8/9) complex formation. These calcium-binding proteins have proinflammatory functions and are potential inflammation biomarkers. More evidence in different fibrosis disorders highlights their role as relevant fibroblast proliferation mediators and prognosis markers. Hence, this review focuses on the current understanding of the role of S100A8, S100A9, and S100A12 calgranulins and calprotectin (S100A8/A9) in the fibrotic process of different disorders, and their potential application as disease severity and prognosis biomarkers.

## 1. Introduction

Fibrosis is characterized by the excessive and progressive deposition of fibrous connective tissue, which usually results in the disruption of the affected tissue or organ architecture. During tissue injury, this process occurs normally during wound healing, but, by a still unknown mechanism, the scarring process is uncontrolled, leading to tissue and organ damage, or even to death. Actually, fibrosis is one of the leading causes of death, accounting up to 45% of worldwide deaths [1]. Fibrosis occurs in diverse tissues and organs, and regardless of the disease etiology, there are prevailing cellular mechanisms that lead to its progression.

The first immune cells recruited to the injured site are neutrophils, which are attracted by chemokines released by activated macrophages. As the inflammatory process progresses, monocytes and lymphocytes are then recruited. Monocytes differentiate into macrophages, which produce additional proinflammatory cytokines and chemokines. If this inflammatory response is acute and chronic, it can lead to epithelial and endothelial cell injury, which in turn triggers the release of inflammatory mediators, such as cytokines and chemokines that stimulate fibroblasts, fibrocytes, myofibroblasts, and fibroblasts derived through an epithelial-to-mesenchymal transition [2]. An intricate molecular network involving an extensive number of molecular pathways, such as the TGF-β/Smad cascade, MAPK pathway, and integrin signaling, among others, in these effector cell subpopulations precedes the deposition of extracellular matrix components (EMCs) that culminates in fibrosis [3]. Among the inflammatory proteins involved in the fibrotic process are some S100 proteins, which regulate calcium homeostasis, as well as the inflammatory process. The S100 proteins are calcium-binding cytosolic proteins with wide regulatory functions, including inflammation, calcium buffering, cell division, apoptosis, cell growth and proliferation, kinase and phosphatase regulation, protein synthesis, and cytoskeleton–membrane interactions, among others [4,5]. There are currently 25 members of the S100 protein family, which share 25–65% of their amino acid sequence, and are low-molecular-weight proteins with similar molecular masses, ranging from 9 to 13 kDa [6]. Although each of the S100 proteins are encoded by an individual gene, members of group A, comprising 19 of the S100 protein members, are encoded within chromosome 1q21, while the other six members (*S100A11P*, *S100B*, *S100G*, *S100P* and *S100Z*) are encoded in different regions [6]. Usually, these calcium-binding proteins are involved in intracellular and/or extracellular regulatory functions, and depending on their functional roles, they are classified in three main subgroups: S100 proteins with only intracellular functions, S100 proteins with intracellular and extracellular functions, and S100 proteins with only extracellular functions [7]. Within the S100 proteins that exert both intracellular and extracellular regulatory effects is the subgroup of S100/calgranulins, comprising S100A8 (also called calgranulin A or MRP8), S100A9 (calgranulin B or MRP14) and S100A12 (calgranulin C) proteins [8]. Since these three S100A proteins bind Ca^2+^ and were found to be highly expressed in granulocytes, they were called calgranulins [8]. Recently, S100/calgranulins are gaining interest as diagnostic and therapeutic targets in inflammatory-associated diseases.

S100 proteins are characterized by a helix-loop-helix motif, also known as the EF-hand motif, containing charged amino acid residues, conferring a high affinity for Ca^2+^, as well as other divalent ions such as Zn^2+^ [9]. These proteins also contain two more EF motifs, one at the N-terminal part and the other at the C-terminal part, and while the first one requires high Ca^2+^ concentrations, a higher Ca^2+^ binding affinity characterizes the last one. These differences in Ca^2+^ binding affinity in the three EF motifs result in protein conformational changes, favoring the heterodimerization among some S100 protein family members [10]. Indeed, an S100A8 homodimer predominantly binds to an S100A9 homodimer, forming a noncovalent heterodimer of ~36.5 kDa called calprotectin (S100A8/9), as this form is more stable compared to the homodimer form of both proteins [11]. Nevertheless, in their homodimer form, calgranulins S100A8 (8.3 kDa) and S100A9 (13.3 kDa) have independent functions of calprotectin (S100A8/9) complex formation [8]. On the contrary, in the presence of Ca^2+^, S100A12 only exists as a homodimer (10.4 kDa) and does not interact with S100A8 or S100A9 [12]. Either as calgranulin or as calprotectin (S100A8/A9), these proteins are involved in the host inflammatory response, mostly during pathogenic microbial infections [13]. This pathogen invasion protection is attributed to divalent cation chelation by calgranulin and calprotectin (S100A8/A9), including Ca^2+^ and Zn^2+^, thus leading to critical changes in signaling pathways that depend on them [14]. Interestingly, these antimicrobial mechanisms also occur in the mucosal epithelia, where, independently of infection, squamous mucosal keratinocytes express both S100A8 and S100A9 to avoid bacterial invasion [15].

This review summarizes the current understanding of the function of S100A8, S100A9, and S100A12 calgranulins and calprotectin (S100A8/A9), focusing on their roles in the inflammation and the fibrotic process in different organ disorders. We highlight their potential application as disease severity and prognosis biomarkers.

## 2. S100A8, S100A9, S100A12 Calgranulins, and Calprotectin (S100A8/A9) in Neutrophils, Monocytes, and Macrophages

**Figure 1:**
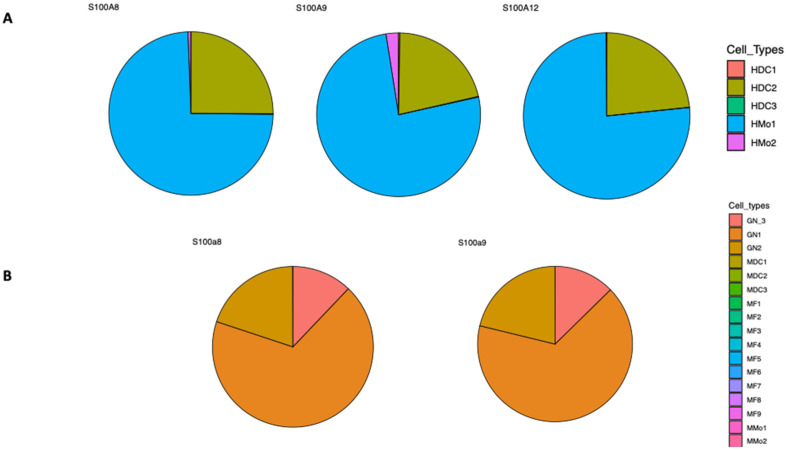
Cell-type-specific expression of calgranulins. (**A**) Expression profiles of human S100A8, S100A9 and S100A12 from the Ultra-low-input RNA-seq (ULI RNA-seq) from the Immunological Genome Project. Human Blood Dendritic cells (HDC1, HDC2, and HDC3) and Human Blood Monocytes (HMo1, and HMo2); (**B**) Expression profiles of mouse S100a8 and S100a9 from ULI RNA-seq (Gene Skyline). Mouse Spleen and Plasmacytoid Dendritic cells (MDC1, MDC2, and MDC3), Mouse Granulocyte (GN1, GN2, and GN3), Mouse Peritoneal Macrophages (MF1–MF9), and Mouse Blood Monocytes (MMo1 and MMo2). Full ImmGen cell-type legend description in Table S1 (https://www.immgen.org/Databrowser19/Expression.html; accessed on 26 January 2026) [28].

Calgranulins are a subgroup of S100 proteins, comprising S100A8, S100A9 and S100A12, that are predominantly expressed in myeloid cells, including neutrophils, monocytes, and macrophages [13,16]. These cell types are among the first immune cells recruited to the site of infection or injury in order to eliminate pathogens, and contain the spread of infection. Within their antimicrobial functions is nutritional immunity, which consists of the sequestration of essential trace metals, such as Zn^2+^, Fe^2+^, and Cu^2+^, therefore restraining the function of essential microbial metalloproteins, including metalloenzymes [17,18]. This mechanism acts as both a host and pathogen dynamic regulator, reshaping immunological responses. Notably, these cells are also mobilized into inflammation areas, regardless if there is a pathogen infection. As the leading immune cell type is recruited to the infection site, neutrophils phagocytose bacteria or fungi, releasing reactive oxygen species (ROS) and antimicrobial molecules [19]. Even if these mechanisms are highly regulated, neutrophil apoptosis triggers macrophage phagocytosis and anti-inflammatory molecule secretion [20]. During the infectious process, this complex host–pathogen interaction also triggers the release of other molecules, including calgranulins S100A8, S100A9 and S100A12, and calprotectin (S100A8/A9), which are highly expressed in these cell subpopulations. While in neutrophils calprotectin (S100A8/A9) constitutes 40–60% of the cytosolic proteins, in monocytes and macrophages it only represents 5%, but upon inflammation it is released with a more than 100-fold increase [13,21,22]. Moreover, upon inflammatory conditions, both S100A8 and S100A9 are expressed in activated keratinocytes, epithelial cells and osteoclasts [13,23,24]. While in the mouse only S100a8 and S100a9 exist, in humans S100A12 constitutes 5% of total cytosolic proteins in resting neutrophils, and is found in a lower extent in monocytes [25]. It has also been detected in the early stage of differentiation of epithelial and dendritic cells [26,27]. Indeed, these calcium-binding proteins exhibit a more subtle cell-type specificity as this is documented in both human and murine expression profiles [28] (Figure 1).

The binding of S100A12 and calprotectin (S100A8/A9) with cell surface receptors, such the multi-ligand receptor of advanced glycation end products (RAGE) and TLR4, triggers the expression of proinflammatory cytokines and the engagement of inflammatory regulation [13,27]. RAGE is a multi-ligand receptor of the immunoglobulin family expressed in leukocytes, promoting cellular activation and migration [28]. This receptor binds to a variety of ligands of diverse origin and molecular properties, recognizing a structural motif rather than a specific ligand [29]. RAGE interacts with S100A12 and calprotectin (S100A8/A9) through the oligomerization of the receptor on the cell surface, and protein structural analyses have revealed that the oligomerization of S100 proteins is also required to bind to RAGE [30,31].

Moreover, the overexpression of RAGE is known to sustain the inflammatory response in some chronic inflammation and age-related diseases through NK-kB activation and the consequent production of proinflammatory molecules [31]. Eventually, tissue damage also leads to a positive-feedback loop in RAGE signaling through the release of other RAGE ligands [32].

During macrophage activation, calprotectin (S100A8/9) translocates to the cell membrane where it localizes with cytoskeleton proteins, suggesting their role in adherence, cell migration, diapedesis, etc. [33,34]. Hence, Ca^2+^ and Zn^2+^ binding by calprotectin (S100A8/A9) is also involved in cell migration, such as in fibrosis. During inflammation, calprotectin (S100A8/A9) is involved in the recruitment of neutrophils as it plays an important role in tubulin polymerization and cytoskeleton rearrangement [35,36]. Non-infectious inflammation triggers common immune responses that involve cell migration, such as fibroblast recruitment to the wound site, which is mediated by proteins like calgranulins and calprotectin (S100A8/A9). Furthermore, it has been described that monocytes can differentiate into fibroblast-like cells called fibrocytes, which share cell markers with leukocytes, hematopoietic progenitor cells and fibroblasts [37]. Interestingly, fibrocytes express extracellular matrix proteins, such as collagen I and III, and vimentin, which are involved in the epithelial-to-mesenchymal transition (EMT) [38,39]. Additionally, fibrocytes have the potential to differentiate into other mesenchymal cells, like adipocytes and myofibroblasts [40], reflecting their cell plasticity to respond to environmental factors. Nevertheless, all these pieces of evidence raise questions into the common cellular and molecular pathways that may occur in the fibrosis process in different organs and tissues.

## 3. Idiopathic Pulmonary Fibrosis

Idiopathic pulmonary fibrosis (IPF) is the prevailing form of pulmonary fibrosis, affecting roughly 3 million people worldwide, with an alarming prevalence increase after the COVID-19 pandemic, probably due to the inflammatory process related to SARS-CoV-2 infection [41,42]. Even if several risk factors have been associated with IPF, such as a genetic predisposition, aging, smoking, constant exposure to dust and air pollution, and working with livestock, among other environmental factors, microbial and viral infections play a crucial role in the outset and exacerbation of IPF [41,43]. Although the specific IPF pathophysiology mechanisms remain unclear, alveolar epithelia micro-injuries, as well as the aberrant activation of epithelial cells, result in the secretion of fibrogenic growth factors, proinflammatory cytokines and chemokines, and an increased vascular permeability to fibrinogen and fibronectin, which eventually form a provisional wound clot in both the interstitial and alveolar spaces [40,41]. In response to these events, alveolar epithelial cells (AECs) are abnormally activated, proliferating and migrating to the injured tissue, and consecutively recruiting resident fibroblasts and fibrocytes by expressing CXCL12, which forms the chemotactic gradient [40,43]. Usually, after initial diagnosis, an IPF patient’s prognosis is poor, with a mean survival time of 35 years [44,45,46]. Therefore, it has been crucial to identify early prognosis biomarkers. For instance, an increased monocyte count has been associated with IPF progression, hospitalization and mortality [47]. Moreover, IPF patients exhibit a differential expression of chemokines, immune signaling proteins, and calgranulins and calprotectin (S100A8/A9), which are involved in signal transduction pathways that perpetuate a proinflammatory and a fibrotic milieu [48]. IPF patients with high levels of circulating calgranulin S100A12 exhibit a lower survival rate, suggesting the effect of the excessive inflammatory immune response mediated by this calgranulin [43]. Interestingly, single-cell RNA-sequencing analysis of lung biopsies of IPF patients revealed that S100A12 is mainly and highly expressed in monocytes [44,45], and it is upregulated both in blood and bronchoalveolar lavage fluids (BALF) [49]. Co-expressing with S100A12 were S100A8 and S100A9, which were negatively associated with lung function [50,51]. Contrary to S100A12, S100A8 and S100A9 were expressed in both monocytes and macrophages, with a higher expression in IPF patients compared to controls, suggesting they might participate in IPF development [52]. Indeed, IPF patients with a higher expression of S100A12 exhibited a more pronounced inflammatory response compared with those with lower S100A12 levels, and that monocytes expressing S100A12 were more likely to differentiate into monocyte-derived dendritic cells [53]. Since during the fibrosis process monocytes participate in fibroblast activation, myofibroblast differentiation, and EMC remodeling, their expression of S100A12 could result in the constant production of proinflammatory cytokines, leading to chronic inflammation.

Moreover, calgranulin S100A9 has been suggested as an IPF biomarker, since it is found to be significantly elevated in BALF from IPF patients and its high serum levels in IPF patients allow us to differentiate them from patients with other interstitial lung diseases [54,55]. This increase in the expression of calgranulins is in line with the primary role of the S100A protein family members to restrain pathogen infection by chelating divalent ions [56,57,58]. Calprotectin (S100A8/A9) plays a crucial role in the inflammatory process and in prompting leukocyte migration in order to contend with infection. In IPF patients, calprotectin (S100A8/A9) participates in cell migration, by activating fibroblast proliferation and their differentiation to myofibroblasts, as well as, producing collagens through RAGE [59,60]. The same RAGE signaling pathway has also been described in human embryo lung fibroblasts, where S100A9 promotes fibroblast growth and proliferation, as well as the secretion of collagen and proinflammatory cytokines [61]. The relevance of calprotectin (S100A8/A9) in fibrosis has been reported in a mouse bleomycin-induced pulmonary fibrosis model, where the inhibition of calprotectin (S100A8/A9) using the anti-S100A8/A9 neutralizing antibody suppressed lung fibrosis progression by blocking the activation of NF-κB in mouse fibroblasts [58]. Moreover, in the fibrotic lesions and healing wounds, along with the resident fibroblasts, monocytes that can differentiate into macrophages are recruited, but also into fibrocytes, which are attracted to the inflammatory lesions, and also participate in tissue repair and in the fibrosis process [59]. Fibrocytes are fibroblast-like cells, and upon TGF-β exposure can differentiate into a myofibroblast-like population expressing α-SMA that is able to contract collagen gels [60]. By identifying cell-specific markers, Pilling et al. were able to differentiate monocyte-derived fibrocytes from monocytes from macrophages and fibroblasts, finding that they express the combination of CD45RO, 25F9, and S100A8/A9. Calprotectin (S100A8/A9) was the only cell marker whose expression was retained after a week of fibrocyte cell culture [37]. Moreover, it has been documented that IPF patients with circulating fibrocytes exhibit a greater mortality risk. It has been shown that fibrocytes play a crucial role in extracellular matrix deposition in diverse focal and diffuse fibrosing disorders related to almost any organ and tissue, including lungs, liver, heart, kidney, and pancreas, among others. These observations raise the question about the pivotal role calgranulins and calprotectin (S100A8/A9) might have in the fibrosis process.

## 4. Cardiovascular Diseases

Cardiovascular disease incidence and mortality rates have drastically increased in the past years, especially in young people [62]. Among the main heart-related problems are myocardial infarction (MI), myocarditis, and atherosclerosis (AS), all related to an inflammatory process. MI causes acute tissue damage, especially due to a substantial necrosis of cardiomyocytes, which triggers an inflammatory response, and consequently, the recruitment of neutrophils and inflammatory monocytes [63,64]. After a while, this proinflammatory response turns to a reparation process, which includes the deposition of scar tissue, and thus, pro-fibrotic conditions occur. Fibroblast activation and proliferation is beneficial in the early stages after MI, but if persistent over time, it shifts into fibrosis. An altered cardiac innate immune response has been observed in individuals with MI or with other heart diseases, including the secretion of proinflammatory cytokines and chemokines, the activation of the inflammasome cascade, and the activation of RAGE and toll-like receptor (TLR) signaling [65,66]. The activation of these immune signaling pathways and myocardial ischemia leads to the production of reactive oxygen species (ROS), mitochondrial dysfunction, apoptosis, necrosis, and autophagy [67].

In AS, the large-medium arteries are subjected to a chronic inflammation caused by the formation of plaques consisting of cholesterol, fatty acids, and cellular debris. The interaction of these plaques with the arterial wall cells and some immune cells, such as macrophages and T cells, cause early lesions that, over time, develop into a more complex necrotic lesion. These lesions are formed by a fibrous cap and the infiltration of mast cells, activated T cells and macrophages, which produce proinflammatory mediators [2,68].

In both acute MI and AS patients, S100/calgranulins are upregulated in damaged cardiomyocytes and fibroblasts, and are secreted by neutrophils, monocytes, and macrophages, such as S100A8 and S100A9 calgranulins, which immediately heterodimerize as calprotectin (S100A8/A9) [68,69]. Transcriptional analyses in MI and atherosclerosis patients have revealed that calprotectin (S100A8/A9) is the most significantly expressed molecule in the early stages of these conditions, and in an S100a9 knockout mouse model, it was shown to be involved in both mitochondrial dysfunction and the inflammatory response [70,71]. In addition, patients with elevated calprotectin (S100A8/A9) serum levels exhibit major adverse cardiovascular events, making calprotectin (S100A8/A9) a valuable candidate for a prognostic biomarker in MI and AS [69,70,72]. In both MI and AS, the increased levels of calgranulins and calprotectin (S100A8/A9) in early stages are firstly produced by neutrophils and macrophages that infiltrate into the infarcted area, which is reflected in the correlation between maximal calprotectin (S100A8/A9) serum levels and maximal blood neutrophil counts in MI patients. After the acute phase of MI, the source of calprotectin (S100A8/A9) is mainly activated macrophages, which finally leads to sustained cardiac fibroblast proliferation [64,69]. Using a murine model, calprotectin (S100A8/A9) was shown to play a detrimental role in the early stage of MI and a short-term blockade of S100A9 calgranulin with the specific blocker ABR-238901 decreases infiltrating neutrophils and macrophages in the ischemic myocardium, decreases cytokine expression in inflammatory macrophages and provides a reparatory microenvironment in the ischemic myocardium [73]. In contrast, long-term blockade (21 days) resulted in progressive deterioration of cardiac function, since it interfered with essential processes for adequate myocardial repair, such as monocyte production, myocardial infiltration, and differentiation into reparatory macrophages [74]. This result highlights the importance of identifying an optimal therapeutic window for S100A9 blockade.

## 5. Liver Fibrosis

Among other organs frequently affected by fibrosis are the liver and the kidneys. Nonalcoholic fatty liver diseases (NAFLDs) are some of the most frequent chronic liver diseases worldwide, and they include nonalcoholic fatty liver (NAFL) and nonalcoholic steatohepatitis (NASH) [75]. NAFLD initiates with steatosis, usually caused by deregulation of lipid metabolism, and it can be exacerbated by insulin resistance, resulting in a more severe disease [76]. Eventually, multiple hepatocyte damage and apoptosis lead to chronic liver inflammation and the recruitment of immune cells, and if this condition persists, it can progress to fibrosis. Concerning the liver, it has been reported that the major precursors of myofibroblasts are hepatic stellate cells (HSCs), which regulate tissue repair by enhancing EMC synthesis and homeostasis, collagen production, chemotaxis, drug detoxification, and activating the inflammatory pathway [77]. Several studies have identified that the activation of HSCs is mediated by multiple signaling pathways, including NF-κB, PI3K/AKT, TGF-β/Smad, MAPK and AMPK, suggesting a significant crosstalk between these pathways involved in liver fibrosis [78,79]. Chang et. al. reported that one of the key proteins that promotes both HSCs and bone marrow-derived mesenchymal stromal cell (BMSC) migration into the damaged liver site is calprotectin (S100A8/A9), overexpressed mostly by neutrophils [80]. A growing body of evidence has proved that liver fibrosis progression can be attenuated or reverted, mostly by targeting S100A proteins and other fibrotic molecules using RNA interference, specific microRNAs, neutralizing antibodies, or knock-down murine models [80,81,82,83]. This feature of liver fibrosis can be attributed to the high cellular plasticity of hepatocytes and other liver cells, which is not present in other organs and tissues. Actually, it has been shown that the removal of the etiological agent reverses hepatic fibrosis [78,83,84]. Interestingly, this effect has also been associated with the inactivation of HSCs, which in turn induces cell senescence and a reduction in collagen synthesis and cell proliferation [85], while neutrophils cause telomere dysfunction in non-immune neighboring cells by oxidative damage [86]. This complex interplay between cell damage, proinflammatory ambient, and aging-related processes poses the question about a possible abnormal cell reprogramming process in fibrosis etiology, including aging hallmarks such as telomere attrition, loss of proteostasis, necroptosis, mitochondrial dysfunction, and epigenetic alterations, among others [87,88].

## 6. Renal Fibrosis

Indeed, similar features have been reported in aging-related renal fibrosis, where infiltrated macrophages exhibit senescence and activation of ferroptosis signaling, thus contributing to chronic low-grade inflammation and EMT [89]. By using a mouse model, Norman et al. have demonstrated that telomere dysfunction in renal fibroblasts leads to the activation of the inflammatory pathway and macrophage to myofibroblast transition (MMT), endothelial to mesenchymal transition (EndMT), and EMT, and ultimately kidney fibrosis [90]. It has been reported that calprotectin (S100A8/A9) contributes to kidney fibrosis through the activation of TLR4 and RAGE receptors, and its overexpression, especially in infiltrated granulocytes, mediates tissue damage and fibrosis through the loss of tubular epithelial cell contacts [91]. Using single-cell RNA sequencing (scRNA-seq) in an acute kidney injury (AKI) murine model, Yao et al., documented that infiltrated macrophages expressing S100a9^hi^Ly6^hi^ are recruited to the injured kidney even before neutrophils, and that this cell subpopulation triggers and expands renal inflammation. In different mouse models of kidney fibrosis, the use of calprotectin (S100A8/A9) inhibitors such as AB38b, paquinimod, and tasquinimod, significantly reduced fibrosis and improved renal function, making calprotectin (S100A8/A9) a reliable target for fibrosis [92,93]. Interestingly, the use of these drugs has the same effect in other fibrotic tissues and organs [93,94,95,96,97,98].

## 7. Autoimmune Diseases

The involvement of both calgranulins and calprotectin (S100A8/A9) in fibrosis has also been described in other conditions, such as neurodegenerative, cancer, and autoimmune diseases [99,100,101,102,103,104,105]. The etiology of these diseases relies on an enhanced and chronic proinflammatory response and an impaired immune response. High calprotectin (S100A8/A9) concentration has been described in different autoimmune diseases such as rheumatoid arthritis (RA), Still’s diseases, and ankylosing spondylitis, among others. For example, in RA, calprotectin (S100A8/A9) is secreted by neutrophils, monocytes and macrophages in the swollen joints, and several studies have reported that its plasma concentration is a reliable biomarker of the expansion of inflammation and the degree of joint damage [106,107,108,109]. In other autoimmune diseases, calprotectin (S100A8/A9) is also highly expressed and serves as a biomarker for both diagnosis and disease prognosis [110,111,112]. For instance, a 12-fold increase in calprotectin (S100A8/A9) in serum levels can be useful in distinguishing patients with active systemic onset juvenile idiopathic arthritis and patients with oligoarthritis, making it a possible biomarker for monitoring disease activity and response to treatment [4]. In RA, synovitis can lead to joint fibrosis, by the hyperactivation and proliferation of fibroblast-like synoviocytes (FLSs), which produce matrix metalloproteinases (MMPs), promoting cartilage inflammation and destruction [113]. Moreover, scRNA-seq studies have revealed that the interaction among synovial fibroblasts and synovial macrophages contribute to RA development, through the excessive production of cytokines and chemokines, leading to fibrosis and hypertrophy of the synovial membrane [114]. Proinflammatory cytokines promote neutrophil and macrophage activation, leading to S100A8 and A9 secretion into extracellular environments, including the synovial fluid of RA patients, showing a 10-fold increase compared to osteoarthritis (OA) patients [115,116]. Moreover, the deletion of S100A8 and S100A9 in an antigen-induced RA murine model resulted in cartilage degradation inhibition, indicating the relevant role of these calcium-binding proteins in the development of RA [117,118,119].

## 8. Neurodegenerative Disorders

Concerning neurogenerative disorders, several studies support a bidirectional relationship between gastrointestinal inflammation and the development of these diseases through the gut–brain axis [120]. More evidence suggests that important changes in the gut microbiota can lead to gut inflammation, resulting in the development of neurodegenerative disorders. High levels of fecal calprotectin (S100A8/A9) have been associated with neurodegenerative diseases such as Alzheimer and Parkinson’s disease (AD and PD, respectively), suggesting its direct effect on gut microbiota composition and intestinal inflammation [101,102,112]. Interestingly, high levels of both S100A8 and S100A9 as monomers and as calprotectin (S100A8/A9) were found in human breast milk, and in the feces of newborns from 30 days to 1 year of age, finding that calprotectin (S100A8/A9) trains intestinal mucosal immunity by influencing the inflammatory response and favoring gut colonization by favorable microbiota [121]. Furthermore, recent studies suggest the involvement of fibrosis in neurodegenerative diseases such as amyotrophic lateral sclerosis, multiple sclerosis and Alzheimer’s disease [122]. The formation of “plaques” in Alzheimer’s disease is due to the extensive deposition of β-amyloid aggregates accumulating with extracellular molecules [123]. Indeed, both patients with Alzheimer’s disease or with ischemic lesions exhibit high levels of calprotectin (S100A8/A9) in the microglia, suggesting their role in the inflammatory and fibrotic processes [124]. In primary rat hippocampal neurons, S100A12 was shown to promote neurite outgrowth through the activation of the MAPK pathway [125]. Other studies have demonstrated increased fecal calprotectin in patients with PD, supporting a bidirectional relationship between gastrointestinal inflammation and PD through the gut–brain axis. More studies are still needed to elucidate the role of these proteins in the fibrotic process in neurodegenerative disease.

**Figure 2:**
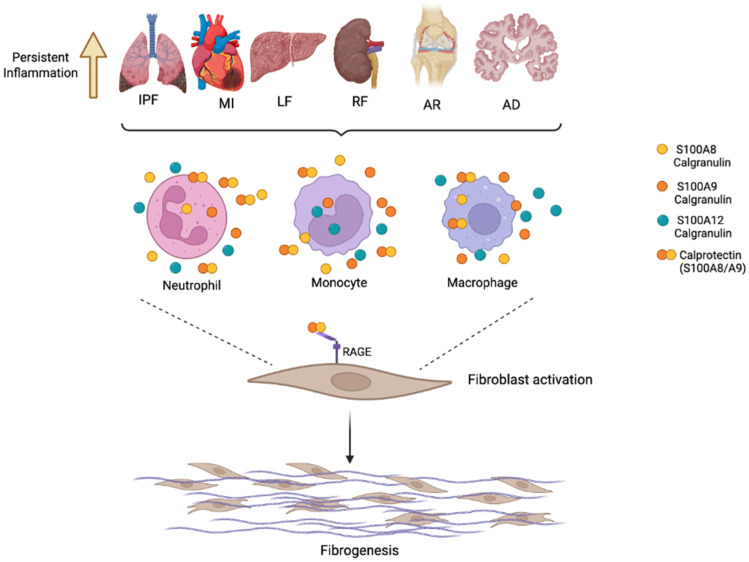
Potential role of calgranulins S100A8, S100A9, S100A12 and calprotectin (S100A8/A9) in inflammation and the fibrotic process in different diseases, such as idiopathic pulmonary fibrosis (IPF), myocardial infraction (MI), liver fibrosis (LF), renal fibrosis (RF), rheumatic arthritis (AR), and Alzheimer’s disease (AD). During the inflammatory process caused by infection, injury or tissue damage, neutrophils, monocytes and macrophages are recruited. Among the inflammatory mediators released by these cell types are calgranulins (S100A8, S100A9, and S100A12), as well as calprotectin (S100A8/A9). These calcium-binding proteins signalize mainly through the receptor for advanced glycation end products (RAGE), inducing fibroblast activation and finally leading to fibrogenesis. Created in BioRender. Ramirez, G. (2026), accessed on 20 February 2026 https://BioRender.com/c5pyzh1.

All these findings imply a broader influence of S100/calgranulins and calprotectin (S100A8/A9) in the regulation of the immune response, in the inflammatory process, and in the etiology of fibrosis (Figure 2 and Table 1).

## 9. Other S100 Proteins Involved in Fibrosis

The involvement of other members of the S100 protein family in fibrosis has been documented in some diseases, where some of them have potential roles as prognosis and disease biomarkers (Table 2).

### 9.1. S100A3/S100A13

S100A3 (S100E) was first described to be highly expressed in hair root cells, having a role in epithelial cell differentiation to hair cuticular barrier formation [6]. In 2019, Al-Mutairy et al. identified two genetic variants in the *S100A3* and *S100A13* genes in two unrelated families with sporadic pulmonary fibrosis, which led to significantly lower S100A3 and S100A13 protein expression, aberrant intracellular calcium responses, reduced tolerance to external oxidative stress and differential expression of ECM proteins [126]. In addition to its role in lung fibrosis, the increased expression of the S100A3 gene has been associated with the development and invasiveness of hepatocellular carcinoma, being a potential target and marker for this disease [127].

### 9.2. S100A4

The S100A4 calcium-binding protein was initially termed fibroblast-specific protein (Fsp1), as it was considered as a fibroblast marker [128]. S100A4, also called calvasculin and metastasin, exert both intra and extracellular functions, but its extracellular function is mainly related to its proinflammatory and pro-metastatic activities [129]. This calcium-binding protein is expressed in different cell types including fibroblasts, inflammatory cells and malignant cells, and has been implicated in the development of fibrosis in IPF, liver, kidney, cardiac hypertrophy and arthritis [128]. S100A4 has been utilized as a marker of EMT in epithelial cells in response to tissue injury [130]. In lung fibrosis, S100A4 is released by M2 alveolar macrophages, enhancing the proliferation and activation of lung fibroblasts through sphingosine 1 phosphate (S1P) [131]. Moreover, S100A4 interacts with cytoskeletal proteins such as topomyosin, actin, and non-muscle myosin II (NMII), participating in cytoskeletal redistribution in fibrosis disorders such as IPF [132]. In the liver, S100A4 promotes liver fibrosis by activating HSCs, and its expression is upregulated during the progression of liver fibrosis, positively correlating with the degree of liver fibrosis [81,133]. S100A4 is expressed in the normal heart tissue, in several interstitial cell types and cardiac monocytes [130]. During cardiac hypertrophy and injury, S100A4 expression is increased in the left ventricles of patients, and localizes to fibroblast-like cells, inflammatory cells and endothelial cells [134].

### 9.3. S100A6

S100A6, also called calcyclin, plays a crucial role in tissue repair and fibroblast proliferation in response to mechanical stress [135]. It is present in most human tissues but it exhibits its highest expression in fibroblasts, epithelial cells, neurons, glia cells and smooth or cardiac muscle [136]. In vitro studies have documented that it regulates lung fibroblast proliferation, morphology, and cytoskeletal organization [137]. High *S100A6* mRNA and proteins levels have been documented in IPF [138], myocardial infraction [139], liver cirrhosis [140], renal fibrosis [141], and neurodegenerative diseases, such as Alzheimer’s disease [142]. In the liver, similar to S100A4, S100A6 contributes to the development and progression of liver fibrosis, by inducing HSC proliferation through RAGE binding and inducing ERK activation [143].

**Table 2:** S100 proteins involved in fibrosis.

S100 Proteins	Alternative Names	Molecular Weight (kDa)	Intracellular/Extracellular Function	Tissue/Cell-Type Expression	Associated Fibrosis Disorder	Clinical Implications	[Refs.]
S100A3	S100E	~12.0	Intracellular	Hair root cells, skin, lung Astrocytomas	SPF	Calcium homeostasis disruption, reduced response to oxidative stress and differential expression of ECM proteins associated with two genetic variants in *S100A3* and *S100A13*	[118]
S100A4	Fsp1/Calvasculin/Metastatin/	12.0	Both	Lung, muscle, colon, skin Fibroblasts, adipocytes, tumor cells, myeloid cells, immune cells	IPF	Enhanced proliferation and activation of lung fibroblasts through S1P; cytoskeletal redistribution by interaction with topomyosin, actin, and NMII	[123,124]
					LF	Activation of HSCs; correlation with the degree of LF	[74,125]
					CH	Increased expression in left ventricles; localization to fibroblast-like cells, inflammatory cells and endothelial cells	[126]
S100A6	Calcyclin	10.1	Both	Brain, heart, lung, muscle, skin Epithelial cells Fibroblasts, tumor cells	IPF, MI, LC, RF, and AD	Elevated mRNA and protein levels	[138,139,140,141,142]
S100A8	Calgranulin A/MRP8	8.3	Both	Neutrophils, monocytes, macrophages, keratinocytes, endothelial cells, epithelial cells	IPF	Highly expressed in monocytes and macrophages; negatively associated with lung function	[44,46]
					MI and AS	Upregulated in damage cardiomyocytes and fibroblasts	[60,61,62,63,64]
S100 A9	Calgranulin B/MRP14	13.3	Both	Neutrophils, monocytes, macrophages, keratinocytes, endothelial cells, epithelial cells	IPF	Highly expressed in monocytes and macrophages; negatively associated with lung function; significantly elevated expression in BALF allows us to differentiate with other lung diseases	[44,45,46,47,48]
					MI and AS	Upregulated in damage cardiomyocytes and fibroblasts	[60,61,62,63,64]
S100A12	Calgranulin C	10.4	Both	Neutrophils, monocytes, macrophages, epithelial cells	IPF	High circulating levels associated with lower survival rate; highly upregulated in lung monocytes, blood and BALF	[44,45,46,47,48]
S100A8/A9 heterodimer	Calprotectin	~36.5	Both	Neutrophils, monocytes, macrophages, keratinocytes, fibrocytes, endothelial cells, epithelial cells	IPF	Highly expressed in monocytes and macrophages; negatively associated with lung function; significantly elevated expression in BALF allows us to differentiate with other lung diseases	[44,45,46,47,48]
					MI and AS	Upregulated in damage cardiomyocytes and fibroblasts; Involved in mitochondrial dysfunction and inflammatory response; correlation with major cardiovascular events	[60,61,62,63,64,65]
					LF	Increased expression during fatty liver injury/fibrogenesis; promotes HSCs and BMSCs migration to the damaged site	[73]
					RF	Contributes to KF through the activation of TLR4 and RAGE receptors	[84]
					RA	In swollen joints secreted by neutrophils, monocytes and macrophages	[7,100,101,102,103]
					ND	High levels of fecal calprotectin are associated with AD and PD	[94,95]
S100A13	--	~11.0	Both	Heart, lung, skeletal muscle, thyroid gland	SPF	Calcium homeostasis disruption, reduced response to oxidative stress and differential expression of ECM proteins associated with two genetic variants in *S100A3* and *S100A13*	[118]
S100A16	S100F	11.8	Both	Adipose tissue, colon, esophagus	RTF	Cytoskeleton reorganization through interaction with myosin-9; Activation of ERS through interaction with GRP78	[136,137]
				Astrocytoma	LV	Increase expression during HSC activation, its deficiency prevents LV	[138]

Fsp1: Fibroblast-specific protein; MRP: Myeloid-related protein; SPF: Sporadic pulmonary fibrosis; IPF: Idiopathic pulmonary fibrosis; LF: Liver fibrosis; CH: Cardiac hypertrophy; MI: Myocardial infraction, LC: Liver cirrhosis; RF: Renal fibrosis; AD: Alzheimer disease; AS: Atherosclerosis; RA: Rheumatoid arthritis; RTF: Renal tubolointerstitial fibrosis; ECMs: Extracellular matrix components; S1P: Sphingosine 1 phosphate; NMII: Non-muscle myosin II; HSCs: Hepatic stellate cells; BALF: Bronchoalveolar lavage fluid; TLR4: Toll-like receptor 4; RAGE: Receptor for advanced glycation end products; PD: Parkinson’s disease; ERS: Endoplasmic reticulum stress; GRP78: Glucose-regulated protein 78.

### 9.4. S100A16

S100A16 is upregulated in several tumors and has also been described as an adipogenesis-promoting factor, negatively regulating insulin sensitivity [4]. Recently, in renal tubulointerstitial fibrosis, S100A16 has also been described to interact with myosin-9, promoting cytoskeleton reorganization [144], and with GRP78 activating endoplasmic reticulum stress [145]. In liver fibrosis, S100A16 expression levels increase during HSC activation, and it has been described that its deficiency prevents liver fibrosis by inhibition of *Cxcr4* expression, suggesting that the inhibition of this calcium-binding protein might be useful for therapeutic strategies [146].

## 10. Conclusions

Fibrosis is involved in a wide spectrum of chronic diseases and frequently is the cause of death. Even if each organ or tissue cell subpopulation mediates this process in a different manner, common pathways are found in the fibrosis processes, such as a proinflammatory environment and cell proliferation and reprogramming. In all of these processes, calgranulins S100A8, S100A9, and S100A12, as homodimers, as well as the heterodimer calprotectin (S100A8/A9), play a pivotal role as crucial signaling molecules in the fibrotic and proinflammatory processes. A considerable body of evidence supports the use of these proteins as potential diagnostic and prognostic biomarkers in a considerable number of diseases. Finally, more detailed studies could bring insights into the mechanisms by which calgranulins and calprotectin (S100A8/A9) proteins transitioned from their antimicrobial functions to proteins that play a significant role in the pathogenesis of fibrosis in multiple tissues and organs.

## Figures and Tables

**Table 1:** Calgranulins S100A8, S100A9, S100A12, and Calprotectin (S100A8/A9) functions in fibrosis disorders.

S100 Protein	Fibrosis Disorder	Biomarker Source	Prognostic	Functional Analysis	[Refs.]
Calgranulin S100A8	IPF	Lung biopsies, BALF, monocytes and macrophages	Lower rate survival, lung dysfunction	--	[44,45]
Calgranulin S100A9	IPF	Serum, lung biopsies, BALF, monocytes and macrophages	Lower rate survival, lung dysfunction, differentiation with other interstitial lung diseases	--	[44,45,46,47,48]
Calgranulin S100A12	IPF	Plasma, lung biopsies, BALF, monocytes and macrophages	Lower rate survival, lung dysfunction	--	[43,44,45,46]
Calprotectin S100A8/A9	IPF	Plasma, lung biopsies, monocyte-derived fibrocytes, murine model	Use as a potential target for IPF therapy	Identification of cell markers involved in monocyte-derived fibrocytes in cell culture derived from lung explants of IPF patients.	[31,52]
	MI and AS	Serum, autopsy, murine model	Most significantly expressed protein in early stages, valuable candidate for prognostic biomarker, correlation with major adverse cardiovascular events	Inhibition of S100A8/A9 in a BLM-induced murine model by using an anti-S100A8/A9 neutralizing antibody	[62,63,64,65,66,67]
				Decreased cardiomyocyte death and heart function improvements in an S100a9 KO murine model (C57BL/6).	
				Short-term and long-term function by using a murine model (C57BL/6) with induced MI by permanent or temporary left coronary artery ligation. The S100A9 blocker ABR-238901 was administrated during the inflammatory phase.	
	LF	Murine model	Use as a potential target for LF therapy	KD of S100A8/A9 using specific siRNA, and injection of S100A9 neutralizing antibody in a murine model (C57BL/6) alleviated liver injury and fibrogenesis	[73]
	RF	Murine model	Use as a potential target for RF therapy	In RF murine models (C57BL/6), the use of calprotectin (S100A8/A9) inhibitors AB38b, paquinimod, and tasquinimod significantly reduced fibrosis and improved renal function	[85,86]
	RA	Plasma, serum, neutrophils, monocytes, macrophages	Use as a reliable biomarker of inflammation expansion and degree of joint damage, and to distinguish RA from other forms of arthritis	--	[7,100,101,102,103,104,105]
	ND	Fecal material	Use as a diagnostic and prognostic biomarker in AD and PD	--	[94,95]

IPF: Idiopathic pulmonary fibrosis; MI: Myocardial infraction; AS: Atherosclerosis; LF: Liver fibrosis; RF: Renal fibrosis; RA: Rheumatoid arthritis; ND: Neurodegenerative disease; BALF: Bronchoalveolar lavage fluid; KO: knockout; AD: Alzheimer disease; PD: Parkinson’s disease; BLM: Bleomycin; KD: Knockdown.

## Data Availability

No new data were created or analyzed in this study. Data sharing is not applicable to this article.

## Supplementary Materials

The following supporting information can be downloaded at: https://www.mdpi.com/article/10.3390/biom16040553/s1, Table S1: Immgen Cell Type Abbreviation Description.
